# Numerical flow experiment for assessing predictors for cerebrovascular accidents in patients with PHACES syndrome

**DOI:** 10.1038/s41598-024-55345-6

**Published:** 2024-03-02

**Authors:** Karol Wiśniewski, Zbigniew Tyfa, Piotr Reorowicz, Michael G. Brandel, Thomas Adel, Damian Obidowski, Krzysztof Jóźwik, Michael L. Levy

**Affiliations:** 1 Department of Neurosurgery, University of California, San Diego-Rady Children’s Hospital, San Diego, CA 92123 USA; 2 Department of Neurosurgery and Neurooncology, Medical University of Lodz, Barlicki University Hospital, Kopcińskiego 22, 90-153 Lodz, Poland; 3 Institute of Turbomachinery, Lodz University of Technology, 219/223 Wolczanska Str., 90-924 Lodz, Poland; 4 Medical University of Vienna, Spitalgasse 23 Str., 1090 Wien, Austria

**Keywords:** PHACES syndrome, Cerebrovascular accident, Predictors, Computational fluid dynamics, Thrombogenic environment, Computational models, Neurology, Risk factors

## Abstract

There is an increased risk of cerebrovascular accidents (CVA) in individuals with PHACES, yet the precise causes are not well understood. In this analysis, we aimed to examine the role of arteriopathy in PHACES syndrome as a potential contributor to CVA. We analyzed clinical and radiological data from 282 patients with suspected PHACES syndrome. We analyzed clinical features, including the presence of infantile hemangioma and radiological features based on magnetic resonance angiography or computed tomography angiography, in individuals with PHACES syndrome according to the Garzon criteria. To analyze intravascular blood flow, we conducted a simulation based on the Fluid–Structure Interaction (FSI) method, utilizing radiological data. The collected data underwent statistical analysis. Twenty patients with PHACES syndrome were included. CVAs were noted in 6 cases. Hypoplasia (p = 0.03), severe tortuosity (p < 0.01), absence of at least one main cerebral artery (p < 0.01), and presence of persistent arteries (p = 0.01) were associated with CVAs, with severe tortuosity being the strongest predictor. The in-silico analysis showed that the combination of hypoplasia and severe tortuosity resulted in a strongly thrombogenic environment. Severe tortuosity, combined with hypoplasia, is sufficient to create a hemodynamic environment conducive to thrombus formation and should be considered high-risk for cerebrovascular accidents (CVAs) in PHACES patients.

## Introduction

PHACEs syndrome was first described in the literature in 1978 and subsequently defined in 1996 by Freiden et al.^1,2^. Its name is an acronym for the symptoms characterizing this group of patients, which include posterior fossa anomalies, hemangioma, arterial lesions, cardiac abnormalities/coarctation of the aorta, and eye anomalies. In 2001, Metry et al. observed an additional symptom contributing to the disease picture, namely sternal malformation^3^. PHACE syndrome is a rare condition with an undetermined prevalence due to the challenging diagnosis and atypical clinical presentation. According to recommendations, investigations for the syndrome should be conducted in cases of infantile hemangioma of the head, newborns without infantile hemangioma but with other features characteristic of PHACE syndrome, and newborns with infantile hemangioma (albeit smaller and with less typical morphology) along with typical changes associated with the syndrome.

One of the most intriguing features of PHACE syndrome is the presence of arterial anomalies, which can include vessel hypoplasia, closure or complete absence of vasculature, persistent fetal circulation, vascular dysplasia, and aberrant origin or course and tortuosity. In 1998, Barrows et al. published a study on a series of 8 patients with PHACE syndrome, where the occurrence of arterial anomalies was associated with a higher frequency of cerebrovascular accidents (CVA), particularly acute ischemic stroke^4^. These results were also confirmed in other studies^5–7^. Although the exact risk of ischemic stroke is unknown, Siegel et al. observed frequent CVAs in this patient group, with the stroke area typically corresponding to the diseased vessel territory^8^. These findings were confirmed in later studies, indicating that the presence of vascular changes is the most significant predictor of ischemic strokes^9^. Interestingly, subsequent studies indicated that the risk of stroke increased especially when patients presented with stenosis larger than 75% or lack of visualization of a main cerebral artery (internal carotid, middle/anterior/posterior cerebral, basilar, and vertebral artery), or no evidence of collateral circulation^10^. Currently, there is no clear explanation for why some patients with arterial abnormalities experience strokes while others do not. It is postulated, that CVA in the PHACES patients population has a multifactorial etiology.

In this report, we looked at the role of arteriopathies in patients with PHACES syndrome to potentially identify a strong predictor for CVA in addition to determining the basic mechanistic underpinnings of our clinical observations. This goal was achieved by means of a statistical analysis followed by computational fluid dynamics (CFD) with a fluid–structure interaction (FSI) method assessment. Numerical simulations were performed utilizing a blood washout approach which will be subsequently described in detail during transient simulations that mimic pulsatile blood flow.

## Material and Methods

### Patients

We performed a retrospective analysis of clinical and radiological data (magnetic resonance angiograms (MRA) and computed topography angiograms (CTA)) of PHACES syndrome patients in the pediatric population. The diagnosis of PHACES syndrome was made by neurosurgeons, neuroradiologists, dermatologists, geneticists and neurologists. We used diagnosis criteria published by *Garzon* et al.^3^. According to the criteria, we stratified two categories:

1. PHACE syndrome, defined by the presence of segmental infantile hemangioma larger than 5 cm on the head, including the scalp, associated with 1 major criterion or 2 minor criteria, or hemangioma of the neck, upper trunk or trunk, and proximal upper extremity PLUS 2 major criteria and,
2. Possible PHACE syndrome, defined by the presence of infantile hemangioma and 1 minor criterion.

**Figure 1:**
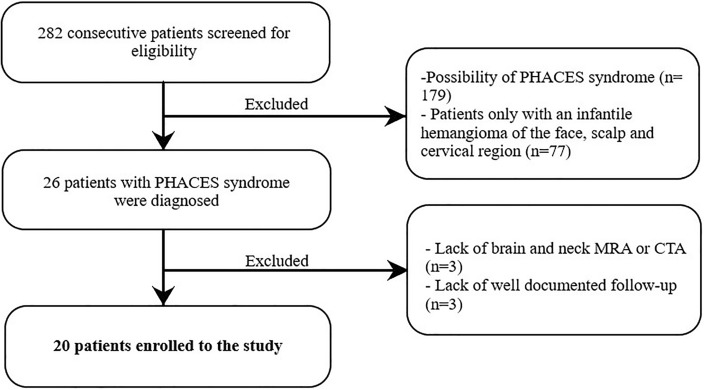
Flowchart presenting the enrollment of the patients.

In this study, we concentrated only on PHACES syndrome not on possible PHACES syndrome. We diagnosed PHACES syndrome in 26 patients. We included to the study only those only patients with head and neck MRA or CTA examinations and with a well-documented follow up visits (after 6-, 12- and 24 months) were included in the analysis. Overall, 20 patients were included in the study analysis (Fig. 1).

CVA was determined by clinical or radiographic signs of cerebral ischemia, including transient ischemic attack (TIA) nonhemorrhagic and hemorrhagic stroke. Diagnosis was confirmed by two independent neurologists.

### Clinical assessment

We analyzed three groups of predictors in our statistical analysis:

1. Clinical factors included:
  age,
  gender.
2. Radiographic factors were analyzed on a routine performed angio-MR and angio-CT using 3D reconstructions and included:
  hypoplasia (of at least one main cerebral artery (internal carotid artery (ICA), vertebral artery (VA), basilar artery (BA), anterior cerebral artery (ACA), middle cerebral artery (MCA), posterior cerebral artery (PCA)),
  absence of at least one main cerebral artery,
  presence of persistent arteries (trigeminal, otic, hypoglossal, proatlantal and primitive ophthalmic artery),
  presence of severe arterial stenosis (greater than 75%) of at least one main cerebral artery,
  presence of severe tortuosity
  presence of saccular aneurysms,
  jugular bulb asymmetry,
  presence of fenestration of superior sagittal sinus,
  presence of brain abnormalities (Dandy-Walker syndrome or unilateral/bilateral hypoplasia of the cerebellum).
3. Other radiographic factors were analyses on angio-MR and angio-CT and included:
  the presence of cardiovascular abnormalities (aortic arch anomaly, coarctation of the aorta, aortic aneurysm, aberrant origin of subclavian artery with or without vascular ring and right-sided aortic arch (double aortic arch).

### Arterial tortuosity

Arterial tortuosity has been defined according to Weibel et al.^12^. The following types of tortuosities were distinguished:

- Kinking—where acute angle is present with an angle of at least 60°
- Looping—when deformation forms a C or S shape created by at least two angles, each of which is a maximum of 90°.
- Coiling—when the vessel makes a 360° turn.

The types of tortuosities were determined in angio-CT or angio-MR. The analysed vessels included the main branches of the artery: the internal carotid artery, the middle cerebral artery, the anterior cerebral artery, the posterior cerebral artery, the basilar artery, and the vertebral artery. Despite several methods proposed in the literature for assessing vascular tortuosity, the reproducibility of results is low^13–15^. Consequently, there is also no classification of tortuosity for individual arteries in the brain that would determine its degree. For this reason, we relied on visual assessment of tortuosity, which had been previously employed in the literature^16^. In order to distinguish between mild and severe tortuosity, the following criteria were established:

- Mild—is characterized by the occurrence of a maximum of two instances of kinking in at least two segments of major arteries, without the presence of looping or coiling.
- Severe (high)—is defined by the presence of more than two instances of "kinking" in at least two segments of major arteries, or the occurrence of looping or coiling.

It is important to note that in the assessment of tortuosity, physiological twists of arteries, such as the siphon of the internal carotid artery, were not taken into account.

### Statistical analysis and sample size analysis

Statistical analysis was performed using the Statistica package (TIBCO Software Inc., CA, USA). The Shapiro–Wilk test was used to evaluate the normal distribution. Continuous variables were reported as means ± SD or median with interquartile range. Nominal variables were analyzed using Fisher’s exact test (n_min_ lower than 5).

Intergroup differences were analyzed using the t-test for normally distributed variables and the Mann–Whitney U test for variables with nonparametric distributions. Stepwise multivariable logistic regression was used and the coefficient of determination (R^2^) was estimated at 0.15. For all analyses, α level was set at 0.05. The Benjamini–Hochberg procedure was used to account for multiple comparisons. Those with a false discovery rate (FDR) lower than 0.20 were selected for multivariable analysis.

We estimated the statistical power of the Fisher's Exact Test through a simulation approach, utilizing the power.fisher.test function from the *statmod* library in R. The key parameters inputted into the simulation were as follows: sample proportions (p1, p2)—based on a single test, sample size (n1 equal to 6 and n2 equal to 14), alpha equal to 0.05, nsim equal to 1000, and alternative equal to "two.sided". Thus, the group composed of 20 patients is sufficient to obtain statistically significant results, especially taking into account the rarity of the disease, it is a good balance between PHACES syndrome incidence and the study power.

### Computational fluid dynamics: blood washout analysis with Fluid–Structure Interaction approach

To assess the influence of PHACES syndrome of the left internal carotid artery (ICA) on blood flow hemodynamics, in-silico investigations of blood flow were performed in four models of systemic circuit vasculature:

- Case #0—reference case study where all arteries were anatomically correct;
- Case #1—left ICA characterized by normal diameter, but high tortuosity;
- Case #2—left ICA characterized by hypoplasticity, but with normal curvature;
- Case #3—left ICA characterized by PHACES syndrome (simultaneous hypoplasticity and high tortuosity).

All analyses were conducted using the Ansys software (Ansys Inc., USA).

#### Modelling of the reference arterial geometry—vessel lumen

By limiting our simulations of blood flow to just a small portion of the systemic circuit (a hypoplastic artery characterized by PHACES syndrome), we would not be able to capture the important phenomena that may occur in other parts of the arterial system. Thus, we decided to perform in-silico investigations in a systemic circuit including the aorta, visceral arteries, lower and upper limb arteries, as well as cerebral vasculature and all afferent blood vessels.

Unfortunately, it was not possible to gather biomedical imaging data of such a large region for a single patient because she/he would be overexposed to harmful radiation. Therefore, we decided to generate entire model by combining several different geometries of smaller regions. A vast majority of the arteries was obtained by analyzing and processing biomedical imaging data, however, depending on the geometry type, varied techniques of model preparation were used. A simplified description of each step of the reconstruction process is presented below:

1. Patient-specific aortic arch with all major branches—reconstruction was based on extraction of the arterial lumen circumferential contours from DICOM images with the use of custom in-house software named AMR (Lodz University of Technology, Poland). Afterwards, the volumetric model was obtained by connecting successive contours with the use of profiles-lofting algorithm implemented in SolidWorks software (Dassault Systèmes, France);
2. Patient-specific descending aorta with visceral arteries up to iliac arteries—reconstruction process was the same as described in point #1;
3. Patient-specific cerebral vasculature together with distal segments of the ICAs and Vas—reconstruction was based on DICOM images segmentation (mainly seed growing algorithm), 3D model extraction and its processing with the use of functionalities embedded in AMR software. Afterwards, 3D surface model was transformed into volumetric one with the use of Auto Skin method implemented in Ansys SpaceClaim module (Ansys Inc., USA) and saved as a file compatible with SolidWorks software;
4. Idealized arteries of lower/upper limbs—the reconstruction was based on data found in the available literature^17^. Based on sagittal and coronal projections of the upper/lower limb arteries, we generated their centrelines. Then, along these centrelines we created ideally circular cross sections of anatomically correct diameters. Finally, the volumetric model was prepared with the use of profiles-lofting algorithm available in SolidWorks program.

All models were merged together with the use of simple Boolean operation available in SolidWorks software. Specialized medical personnel analyzed the final geometry in terms of anatomical correctness and approved its further use for this research.

#### Modelling of the reference arterial geometry—vessel walls

Fluid–structure interaction (FSI) simulations require having the structural (mechanical) model apart from traditional fluid domain. In our case, such a model should mimic the vessel walls that are characterized by vasomotion, expanding during systole (absorbing energy) and vasodilating during diastole (reverting to their initial size and returning part of the absorbed energy to the fluid to make it flow continually). We used SolidWorks to generate walls of anatomically correct thickness by offsetting surfaces of the volumetric object by a specific distance corresponding to the desired thickness. We encountered problems with offset surfaces overlapping or impossible-to-solve topology in areas where two arteries (or fragments of the same artery) were passing near each other in extremely close proximity. This was resolved by projecting 3D splines onto desired surfaces, remove overlapping regions and creating a tangent connection between both structures. Moreover, traditional hole-filling with tangent constraints was usually insufficient, so one had to generate several guide curves and fill the interior with tangent patches.

#### Modelling of the reference arterial geometry—porous bodies

In CFD analyses of blood flow, flow directioning occurs due to pressure gradient between inlet and outlet cross sections. Pressure drops result from flow resistances. As pressure increases at an outlet cross section, less fluid will reach that region and more fluid will instead pass through the other channels with lower resistances. Therefore, we used porous bodies to provide artificial resistance behind the original outlet cross sections and control flow distribution across the entire systemic circuit. By modulating resistance in porous bodies we could obtain desired pressure drops or increases (see section "CFD with FSI approach—initial validation and investigated parameters". CFD with FSI approach—initial validation and investigated parameters). The longer the porous body the higher the resistance, with the length equal to the circumference of the corresponding outlet cross section. In our case, the length of the porous body was always equal to the circumference of the corresponding outlet cross section.

#### Modelling of the arteries under investigation (including PHACES syndrome)

**Figure 2:**
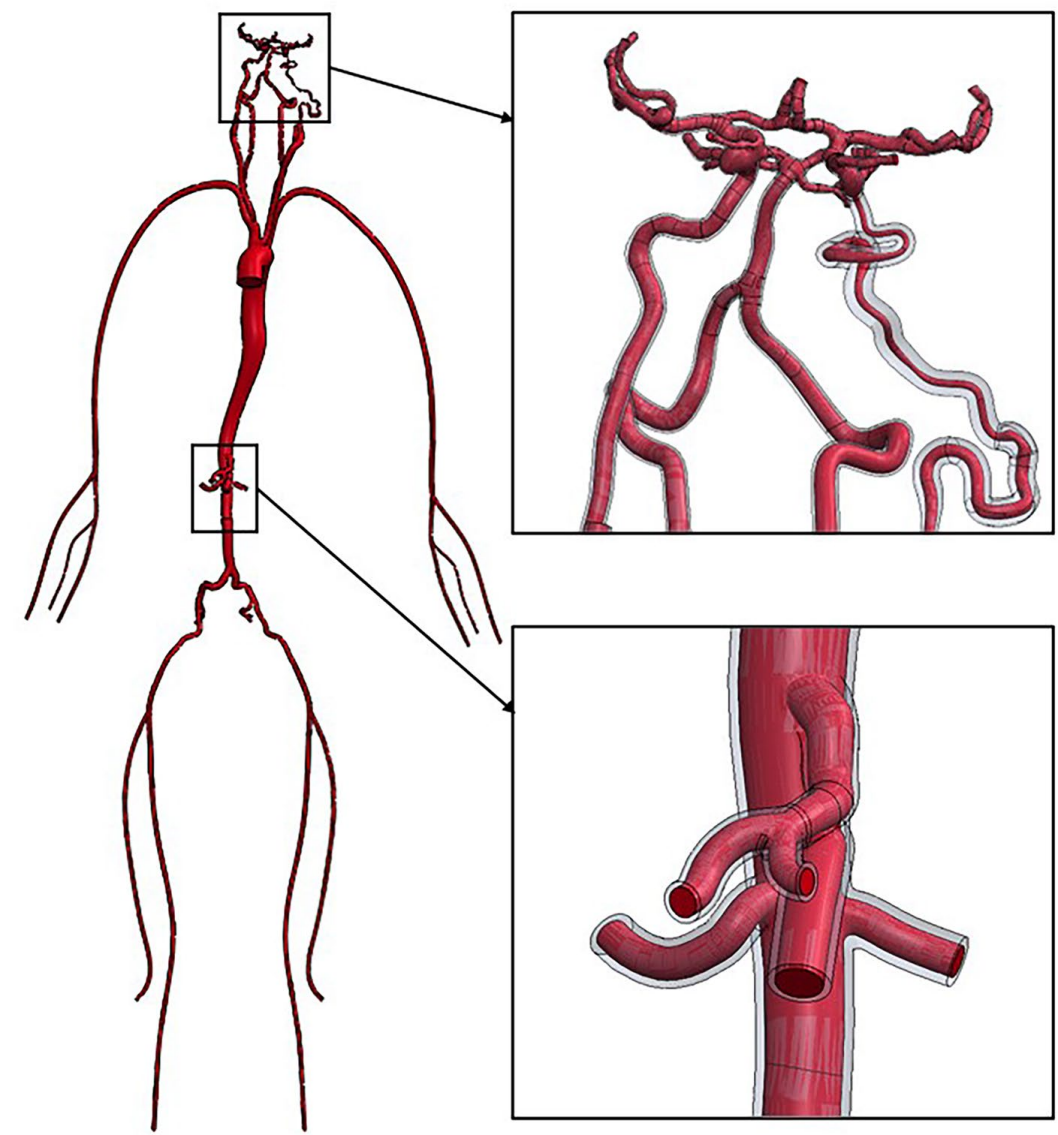
Geometry of the systemic circuit used during this study. PHACES syndrome is visible in one of the internal carotid arteries. Walls are made transparent, while vessel lumen is marked with a red color.

**Figure 3:**
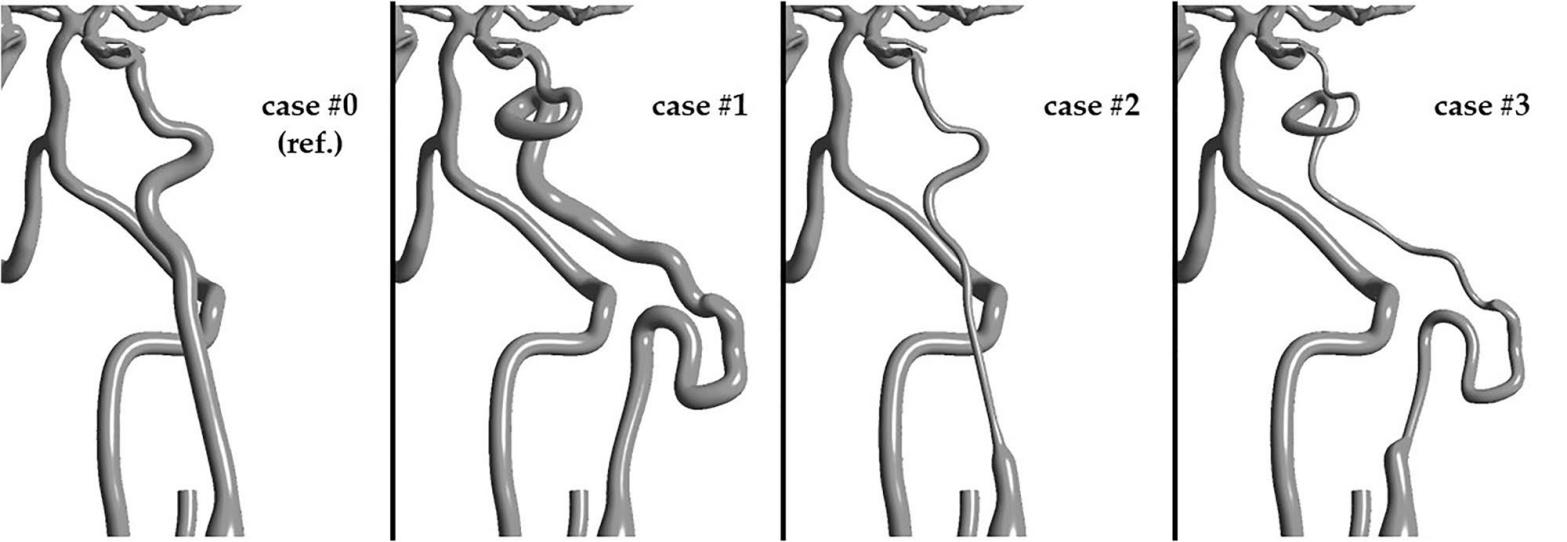
Comparison of all geometries: case #0—reference geometry (normal diameter, normal tortuosity); case #1—left ICA with normal diameter and high tortuosity; case #2—left ICA with hypoplasia, but normal tortuosity; case #3—PHACES syndrome (hypoplasia + high tortuosity).

Due to the absence of a patient-specific DICOM dataset of a patient with PHACES syndrome, we artificially generated the hypoplastic and tortuous ICA for this model. We loaded JPEG images of sagittal and coronal projections of the ICA changed with PHACES syndrome (images found in the online database: https://radiopaedia.org/cases/phace-syndrome-1) and used the same algorithm as previously described for the upper/lower limbs arteries. We next substituted the patient-specific ICA with this artificial one. Models in cases #2 and #3 were generated in the same manner. The entire model of the systemic circuit is presented in Fig. 2. Figure 3 depicts the cerebral vasculature and afferent blood vessels for all cases.

#### CFD with FSI approach—numerical procedure

The physiological conditions of the cardiovascular system can be mimicked using FSI. The two-way coupling algorithm inside the numerical solver allows force generation (deriving from the fluid motion and pressure) that leads to the displacement of the model wall and vice versa. As a result, deformation of the wall affects flow. This approach simulates vasomotion of the arteries, their pressure-damping properties, and sustaining the continual forward flow of blood. Those simulations usually are limited only to a part of an artery, due to their high complexity and time-consuming nature. In this study, we performed analyses of the whole brain arterial vasculature and afferent arteries to assess blood flow stagnation which is a known component of arteriopathies.

#### CFD with FSI approach—numerical mesh

Ansys Fluent module (Ansys Inc., USA) was used to generate the volumetric mesh of the fluid domain, which comprised polyhedral elements together with prismatic ones embedded in an inflation layer near the wall. The inflation layer is mandatory to investigate any phenomena occurring near the walls with suitable precision. The number of sublayers within the inflation layer influences the obtained results in the vicinity of the walls (more sublayers, better flow approximation). In the case of FSI simulations we had to limit their number to 8, since more sublayers would result in their improper deformation (negative volumes) which would result in numerical solver failures—it is a balance between numerical data certainty and possibilities of mesh errors occurrence. The growth ratio between successive sublayers was equal to 0.278. The fluid mesh in our study consisted of circa 4.3 million elements and 10.7 million nodes. The volumetric mesh of arterial walls was prepared in Ansys Transient Structural module (Ansys Inc., USA) with the use of the Linear Order discretization scheme. We decided to generate a mechanical mesh composed of just a single layer of irregular tetrahedral SOLID285 elements (3D lower order elements characterized by 4 nodes) to reduce the computation process time. Finally, the mechanical mesh consisted of circa 1 million elements and 0.28 million nodes.

#### CFD with FSI approach—physical properties of fluid

We assumed no heat exchange and constant temperature of the fluid (since heat exchange occurs primarily in capillaries, not arteries). We assumed that blood density could vary within the range of 1053–1056 kg/m^3^. Otherwise, for fully incompressible fluid, calculations had a high risk of failure due to solver instability. Non-Newtonian shear-thinning behavior was included among the rheological parameters of simulated blood. This means that viscosity decreased with an increasing shear rate, following its specific morphological response. Formed elements of blood, mainly red blood cells, start to deform during medium and high shear rates, and the blood viscosity decreases. When the blood velocity decreases, the shear rates decrease as well, while cells start to form larger aggregations leading to an increase in viscosity. In the literature, there are several dozen mathematical descriptions of blood rheology as it depends on multiple patient-specific variables (sex, age, diet, etc.). For our analyses, we chose a modified power law, as was also proposed in other CFD^18–21^. Equations for our blood model are provided below:

1 $$\left\{\begin{array}{c}\begin{array}{ll}\eta =0.55471 \mathrm{Pa s}& {\text{for}} \dot{\gamma }\le 0.001 \\ \eta = {\eta }_{0}\cdot {\left(\dot{\gamma }\right)}^{n-1}& {\text{for}} 0.001 \le \dot{\gamma }<327\\ \eta =0.00345 \mathrm{Pa s}& {\text{for}} \dot{\gamma }\ge 327\end{array}\end{array}\right.$$

where ${\eta }_{0}$ = 0.035 kg m^−1^∙s^−1.4^; $n$ = 0.6; while $\eta$ = 0.00345 Pa s is a reference viscosity of Newtonian blood.

#### CFD with FSI approach – physical properties of the numerical domain

**Figure 4:**
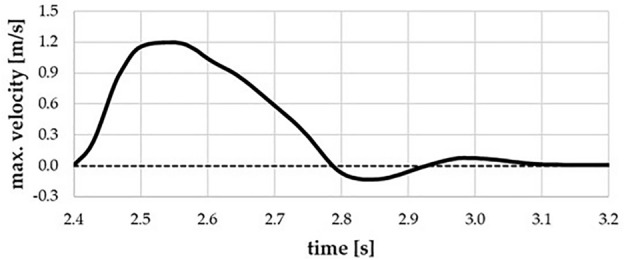
A fragment of the boundary condition imposed on the inlet cross section—the figure presents data for a single cardiac cycle.

To define regions prone to blood stagnation, we investigated the blood washout phenomenon. The washout analysis relies on the principle that at a time equal to 0 s, ‘old blood’ occupies an entire volume of the fluid domain and the blood velocity equals 0 m/s. Subsequently, ‘new blood’ starts to flow inside the numerical domain, pushing ‘old blood’ out of the domain accordingly to the specification of the inlet boundary condition. We set no mixing of the two separate fluids (inside a single mesh element), so there should be either ‘old’ or ‘new blood’. After each cardiac cycle, the remaining blood volume in the altered ICA models was compared to the reference model. Twenty full cardiac cycles were simulated to ensure that (1) initial conditions did not influence the final results, and (2) a sufficient amount of ‘old blood’ was replaced with ‘new blood’. Flow parameters and wall deformations estimated for each cardiac cycle achieved repeatability after 5–6 cycles. At the inlet cross-section to the ascending aorta, a time-varying boundary condition was set by Prandtl profile distribution formula—see Eq. 2. Specific user-defined function (UDF) file was developed and imported into Ansys Fluent to correctly assign the time-dependent velocity function together with profile distribution constrains (oriented normally to the inlet surface). Figure 4 depicts the time-dependent function of the maximal velocity (*V*_*max*_) at the inlet cross section, limited to a single cardiac cycle for visualization purposes.

2 $${V}_{p}={V}_{max}\cdot {\left(1-\frac{r}{{R}_{max}}\right)}^{0.8}$$

We used the same boundary conditions at each outlet cross-section (located behind porous bodies): pressure was initially set as 0 Pa, and linearly increased to 6 kPa after 0.2 s; it maintained this constant value for the rest of the simulation. This was required to perform an ‘initial pumping’ of the fluid domain. Artificial resistances of porous bodies, that helped in maintaining the proper flow distribution to specific regions of the systemic circuit, were controlled with two resistance factors: viscosity and inertia. Their values depended on the region of the cardiovascular system. Both resistance factors can be calculated from Ergun’s equation which describes a total pressure drop through a porous medium—see Eq. (3).

3 $$\frac{\Delta p}{L}=\overrightarrow{U}\left(\frac{150\mu }{{D}_{p}^{2}}\cdot \frac{{(1-\varepsilon )}^{2}}{{\varphi }^{2}{\varepsilon }^{3}}\right)+{\overrightarrow{U}}^{2}\left(\frac{1.75\rho }{{D}_{p}}\cdot \frac{\left(1-\varepsilon \right)}{{\varphi \varepsilon }^{3}}\right)$$

where $\Delta p$—pressure drop, $L$—porous body length, $\overrightarrow{U}$—fluid velocity, $\mu$—fluid dynamic viscosity, ${D}_{p}$—spherical diameter of particles ‘forming’ the porous body), $\varepsilon$—porosity of the body, $\varphi$—sphericity, $\rho$—fluid density.

We assumed $\varphi$ equal to 1.0, ${D}_{p}$ equal to 0.5 mm and porosity $\varepsilon$ changing from 100 to 69% during first 0.8 s. This approach was mandatory to maintain the solver stability during the first cardiac cycle. All other parameters were dependent on the location of the porous body. For example, length of the porous body, $L$, was always equal to a circumference of the corresponding outlet cross section. After a few mathematical modifications of the Ergun’s equation (by comparing it with Ansys Fluent equation for a momentum sink—Eq. 4), we could estimate both viscous and inertial resistances, ${R}_{v}$ and ${R}_{i}$, respectively—see Eqs. (5) and (6).

4 $$\frac{\Delta p}{L}={R}_{v}\mu \overrightarrow{U}+\frac{1}{2}{R}_{i}\rho {\overrightarrow{U}}^{2}$$

5 $${R}_{v}=\frac{{150(1-\varepsilon )}^{2}}{{\varphi }^{2}{{D}_{p}^{2}\varepsilon }^{3}} [{{\text{m}}}^{-2}]$$

6 $${R}_{i}=\frac{2\cdot 1.75\cdot \left(1-\varepsilon \right)}{{{D}_{p}\varphi \varepsilon }^{3}} [{{\text{m}}}^{-1}]$$

The final boundary conditions for the outlet cross sections included the following factors: viscous resistance within the porous domain, inertial resistance within the porous domain and constant pressure equal to 6 kPa. As stated before, both flow resistance factors depended on the location of the corresponding porous body in the domain. Table 1 stores information on these parameters, where ${R}_{v}$ and ${R}_{i}$ correspond to the base values which could be calculated from Eq. (5) and Eq. (6). For the calculation settings in Ansys Fluent solver, we utilized SIMPLE algorithm that belongs to pressure–velocity coupling.

**Table 1:** Viscous and inertial resistance factors in porous bodies located in specific regions of the fluid domain.

Region	Body resistance factors	
cerebral arteries	1.80∙${R}_{v}$	1.80∙${R}_{i}$
external carotid arteries	2.03∙${R}_{v}$	2.03∙${R}_{i}$
viscera arteries	1.60∙${R}_{v}$	1.60∙${R}_{i}$
upper limb arteries	1.80∙${R}_{v}$	1.80∙${R}_{i}$
lower limb arteries	2.80∙${R}_{v}$	2.80∙${R}_{i}$

For the mechanical solver settings, we assumed that mechanical properties of the walls should be governed by neo-Hookean formula of hyperelastic material with the initial shear modulus set to 0.2 MPa and incompressibility parameter set to 10^7^ Pa^−1^. For the calculation purposes a *Large Deformation Option* was chosen. To prevent any unwanted movement or displacement of the outlet/inlet surfaces, which could lead to solver instability, we fixed these patches in 3D space. The remaining regions of the walls could deform while being subjected to external forces and their displacement was limited by elastic support (foundation stiffness coefficient, FS—that mimicked the presence of surrounding tissues) and external pressure acting on the model walls. Initially, pressure value was set as 0 Pa, but it linearly increased to 5 kPa within 2.4 s to become constant for the rest of the simulation. Timestep defined in the mechanical solver was the same as for the Fluent solver − 0.016 s.

#### CFD with FSI approach—general settings

We analyzed 20 full cardiac cycles. Each cycle was characterized by a period of 0.8 s and a timestep of 0.016 s (50 timesteps within a single cycle). We used k-ω Shear Stress Transport (SST) turbulence model which is the gold standard for biomedical flow analyses. We assumed two-way coupling, i.e. mutual interaction of mechanical wall and fluid structure. The internal surface of the mechanical wall is simultaneously an external surface of the fluid domain.

We set the minimum number of iteration loops as 5 within the coupling module (fluid and mechanical solvers exchange information to fulfill two-way coupling algorithm). When either the residuals value dropped below 10^–2^ or when 20 iterations were performed, the coupling computation process was treated as completed. The convergence criteria for the Fluent solver were set as follows: the maximum number of iterations—15; convergence criteria—10^–3^ (absolute) and 5∙10^–2^ (relative). We allowed for 22 iterations of the Transient Structural solver after which it decreased the timestep until the proper solver stability was reached. The “remeshing” option was activated (initiated after calculating 5 timesteps) to smooth vertex positions.

#### CFD with FSI approach—initial validation and investigated parameters

Prior to conducting target numerical simulations, validity and reliability of CFD-generated data were confirmed. In terms of the mesh quality, the mesh was characterized by max. skewness equal to 0.85 at the surface and 0.95 in an entire volume. Pressure and blood flow distribution were analyzed for the reference geometry (case #0). Since physiologically correct values were obtained for all parameters, the proposed FSI settings suitably mimicked overall flow hemodynamics in the systemic circuit—see Table 2.

**Table 2:** Chosen hemodynamic parameters used during verification of the CFD results correctness.

Total volume of delivered blood	Brain region	Neck and face region	Upper limbs region		Abdominal viscera and lower limbs
Blood distribution across the chosen regions of systemic circuit [cm^3^]					
83.76	14.01 (16.7%)	4.32 (5.2%)	8.28 (9.9%)		57.15 (68.2%)
Systole/diastole pressure at model walls		Systole peak		16.7 kPa (125 mmHg)	
		Diastole end		11.5 kPa (86 mmHg)	

During the results analysis, we focused on multiple hemodynamic parameters, including normal stress (pressure) and tangent stresses (wall shear stress, WSS), time-averaged WSS (TAWSS) and oscillatory shear index (OSI) at the ICA walls. Moreover, we investigated blood supply, relative ‘old blood’ area at the control surface (which could be calculated only by simulating blood washout phenomenon) as well as blood viscosity at this surface (since thrombus formation might be initiated when viscosity is elevated).

### Ethical approval and patients electronic data securing

This study was approved by the institutional review board of the University of California, San Diego, which waived the requirement for informed consent. The IRB number (120518), date 20.12.2023–15.01.2024. Electronic medical records were reviewed from Rady’s Children Hospital for all patients with PHACES syndrome who underwent MRI from 2005 to 2022. All patients were evaluated in the pediatric neurosurgery clinic or pediatric dermatology clinic for cutaneous vascular anomalies. All experiments were performed in accordance with relevant guidelines and regulations. The study was designed in accordance with the Good Clinical Practice (GCP) guidelines and was conducted according to the principles of the Declaration of Helsinki.

Patients’ data was stored in electronic form in password protected. Monthly Raw data backup was administered by data servant. Access to all devices used to generate data and storage was controlled at the user level using standard MS Windows domain protocols. Sensitive data was collected in a “as-few-as-necessary” manner. All sensitive data was password-protected in addition to specifically defining user -level access. Passwords were distributed using other communication channels than used for the data transfer.

## Results

### Patients

A total of 282 patients were screened for eligibility and 20 were included (Fig. 1). The median age was 60 months (interquartile range [IQR] 15.25–132) at the time of analysis. There were 15 females and 5 males. We observed CVA in 6 patients. The presenting signs were seizure (3 cases) and transient hemiparesis (3 cases). The median age at time of stroke was 13.5 months (10–49.5). In all CVA cases, cerebrovascular imaging was performed. We did not note hemorrhagic stroke. Mostly, we observed small ischemic strokes in the MCA distribution. None of the children in our study had sequelae of CVA at a 2 year follow up*.* Individual patient characteristics are listed in Table 3.

**Table 3:** Patients clinical data.

Age (months)	Gender	CVA	Hypoplasia	Mild tortuosity	Severe tortuosity	Absence of at least one main cerebral artery	Presence of persistent arteries	Presence of severe arterial stenosis (larger than 75%)	Presence of saccular aneurysm	Presence of brain structural abnormalities	Presence of cardiovascular abnormalities	Jugular bulb asymmetry	Presence of fenestration of superior sagittal sinus
192	F	0	0	0	1	0	1	1	0	0	1	1	0
228	F	0	1	1	0	0	0	0	0	1	0	1	0
168	F	0	0	0	0	0	0	0	0	0	0	0	0
48	F	0	0	0	0	1	1	1	0	1	1	1	1
60	M	0	1	0	0	1	0	1	1	0	0	0	0
6	F	1	1	0	1	1	1	0	0	1	1	0	0
156	F	0	0	0	0	0	0	1	0	0	0	0	0
60	M	1	1	0	1	1	0	1	0	0	0	1	0
10	M	1	1	0	1	0	1	0	0	0	1	0	1
132	F	0	0	1	0	0	0	1	0	1	0	0	0
96	F	0	0	0	0	0	0	0	1	0	0	1	0
17	F	1	1	0	1	1	1	0	0	0	0	0	0
60	F	1	0	1	0	1	1	1	1	1	0	1	1
6	F	0	1	0	0	0	0	0	0	0	1	0	0
6	F	0	0	0	0	1	0	0	1	0	0	0	0
36	M	0	0	0	0	0	0	1	0	1	1	1	0
132	F	0	1	0	1	0	0	0	1	0	1	0	1
72	F	0	0	0	0	0	0	1	0	0	0	0	0
108	F	0	0	0	0	0	0	0	1	1	0	1	0
10	M	1	1	0	1	1	0	1	0	1	1	1	1

### Statistical analysis

In the first step, we performed the univariate analyses comparing patients with and without CVA. Our analyses revealed significant differences between rates radiological risk factors. The CVA incidences were more likely to happen if one of the following risk factors appeared: hypoplasia (p equal to 0.03), absence of at least one main cerebral artery (p equal to 0.009), presence of persistent arteries (p equal to 0.01) and severe tortuosity (p equal to 0.03). Details are presented in Table 4.

**Table 4:** Patient characteristics by CVA status. The results of univariate analysis.

Variable	With CVA	Without CVA	*p* value
Age (years)	8 (5.25–10)	10 (5–14.5)	0.06^a^
Gender	M = 3/F = 3	M = 2; F = 12	0.232^b^
Hypoplasia	n = 5 (6)	n = 4 (14)	0.03^b^
Mild tortuosity	n = 1 (6)	n = 2 (14)	0.680^b^
Severe tortuosity	n = 5 (6)	n = 2 (14)	0.007^b^
Absence of at least one main cerebral artery	n = 5 (6)	n = 3 (14)	0.01^b^
Presence of persistent arteries	n = 4 (6)	n = 2 (14)	0.03^b^
Presence of severe arterial stenosis (larger than 75%)	n = 3 (6)	n = 7 (14)	0.685^b^
Presence of saccular aneurysm	n = 1 (6)	n = 5 (14)	0.387^b^
Presence of brain structural abnormalities	n = 3 (6)	n = 5 (14)	0.642^b^
Presence of cardiovascular abnormalities	n = 3 (6)	n = 5 (14)	0.455^b^
Jugular bulb asymmetry	n = 3 (6)	n = 6 (14)	0.574^b^
Presence of fenestration of superior sagittal sinus	n = 3 (6)	n = 2 (14)	0.131^b^

*P* values calculated using ^a^t-student test and ^b^Fisher exact test.

Subsequently, we performed the multivariate analysis to find the most important clinical and radiological predictor for CVA in PHACES syndrome. To this analysis we included only variables that were significant on univariate analysis. In our research, severe tortuosity was the most important positive predictor of CVA in PHACES syndrome (OR equal to 30, 95% CI 2.2–411.0, p equal to 0.01). Its value was significantly greater in all patients with CVA.

### Computational fluid dynamics: blood washout analysis with Fluid–Structure Interaction approach

Firstly, we analyzed blood flow supply through the chosen regions of the systemic circuit. It turned out that different geometry of the left ICA (Internal Carotid Artery) led to significant changes in blood flow through this vessel (Table 5).

**Table 5:** Blood flow distribution across entire systemic circuit—comparison of all investigated case studies.

Artery	Blood volume distributed in a full cycle [cm^3^]			
	Case #0 (ref.)	Case #1	Case #2	Case #3
Aorta inlet	64.08	64.20 (0.2%)	64.11 (0.0%)	64.09 (0.0%)
Left anterior lobe	2.07	1.96 (− 5.3%)	1.77 (− 14.5%)	1.67 (− 19.1%)
Left posterior lobe	1.70	1.67 (− 2.1%)	1.72 (0.7%)	1.74 (2.0%)
Right anterior lobe	1.34	1.34 (− 0.3%)	1.45 (8.1%)	1.50 (12.1%)
Right posterior lobe	1.23	1.22 (− 1.3%)	1.27 (2.5%)	1.29 (4.2%)
Left vertebral artery (VA)	1.77	1.91 (7.9%)	2.23 (25.7%)	2.38 (34.2%)
Right vertebral artery (VA)	1.38	1.45 (4.8%)	1.69 (21.9%)	1.80 (30.0%)
Left ICA (pathological)	3.82	3.00 (− 21.6%)	1.21 (− 68.3%)	0.44 (− 88.5%)
Right ICA	2.96	3.25 (9.7%)	4.18 (41.1%)	4.54 (53.0%)
Left external carotid artery (ECA)	1.76	1.75 (− 1.0%)	1.86 (5.2%)	1.90 (7.5%)
Right external carotid artery (ECA)	2.01	2.00 (− 0.5%)	2.11 (5.2%)	2.15 (6.9%)
Visceral arteries	22.26	22.71 (2.0%)	21.91 (− 1.6%)	21.88 (− 1.7%)
Upper limbs arteries	6.75	6.65 (− 1.5%)	7.03 (4.2%)	7.19 (6.5%)
Lower limbs arteries	6.90	6.85 (− 0.6%)	7.35 (6.6%)	7.52 (9.0%)

Blood delivered throughout an entire cardiac cycle dropped from 3.82 cm^3^ for the refence geometry to 3.00 cm^3^ for the geometry of normal diameter and high tortuosity, to 1.21 cm^3^ for the hypoplastic artery without severe tortuosity vessel, and to merely 0.44 cm^3^ for the ICA affected by PHACES syndrome. Individual anomalies in vessel course and caliber resulted in substantial flow intensity reductions (21.6% for tortuosity and 68.3% for hypoplasticity). However, simultaneous alterations of the blood vessel (high tortuosity + hypoplasticity) reduced blood flow by 88.5%.

Blood supply reduction to the left ICA resulted in an increase of blood delivery to the right ICA and bilateral VAs in a stepwise fashion. However, upregulation in blood flow of the other main cerebral arteries was insufficient to reach the total baseline bilateral cerebral blood flow in case #0. In case #1, total anterior circulation cerebral blood flow was reduced by 5.3%, while the highest discrepancies were noted for PHACES syndrome case study—over 19% for the left anterior lobe and over 12% for the right anterior lobe.

Differences were also seen for extracranial arteries. There was a slight decrease in blood flow within both ECAs as well as the arteries of the upper and lower limbs for case #1—from 0.5 to 1.5% blood supply reduction. In case #2, most extracranial arteries had a small increase in flow intensity (maximum 6.6% increase for lower limb arteries). In case #3, flow increases were larger than case #2, reaching 9.0% in the lower limbs.

We also analyzed how ICA geometry affects possible stagnation zones (blood velocity lower than 0.01 m/s), blood washout phenomenon, and corresponding blood viscosity. We estimated relative blood stagnation area at control surface being an offset of 0.5 mm from the ICA artery wall. We based this analysis on the surface that was shifted slightly inwards the domain due to an imposed boundary condition at the wall, i.e. *No Slip*. It forces the solver to set velocity at walls to a value of 0 m/s. Data concerning these three parameters are outlined in Table 6.

**Table 6:** Values of the chosen hemodynamic parameters at the wall of left ICA (syndrome-affected).

Parameter		Syndrome-affected artery region			
		Case #0 (ref.)	Case #1	Case #2	Case #3
Systole peak (t at 15.52 s)	relative blood stagnation area at control surface	0.03%	0.16%	5.69%	13.86%
	relative remaining ‘old blood’ area at control surface	0.05%	0.20%	2.07%	27.18%
Late diastole (t at 16.00 s)	relative blood stagnation area at control surface	0.10%	2.02%	13.39%	18.31%
	relative remaining ‘old blood’ area at control surface	0.06%	0.17%	1.04%	27.09%
	maximal dynamic viscosity [mPa s]	23.27	24.42 (4.9%)	21.05 (− 9.6%)	39.00 (67.6%)
	area-averaged dynamic viscosity [mPa s]	4.02	5.00 (24.5%)	5.66 (40.9%)	7.13 (77.5%)

**Figure 5:**
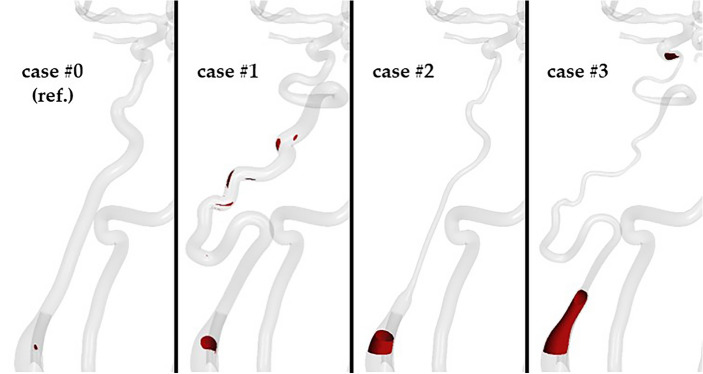
Blood stagnation (velocity lower than 0.01 m/s) areas for each analyzed case studies at the final timestep of the simulation (*t* at 16.00 s).

Blood stagnation followed a trend similar to the blood flow, with the smallest difference compared to baseline for ICA of normal diameter and high tortuosity, and the largest for ICA characterized by PHACES syndrome. In the reference case, almost no regions of stagnant blood or ‘old blood’ were present, whereas for the PHACES syndrome artery stagnation was seen at almost 14% of the control surface and over 27% of its region was covered by ‘old’ not-washed-out blood. A qualitative comparison of blood stagnation is depicted in Fig. 5 (for the final timestep *t* at 16.00 s) and since blood velocity during diastole is reduced compared to systole, there was a greater degree of blood stagnation in diastole compared to systole for each case.

**Figure 6:**
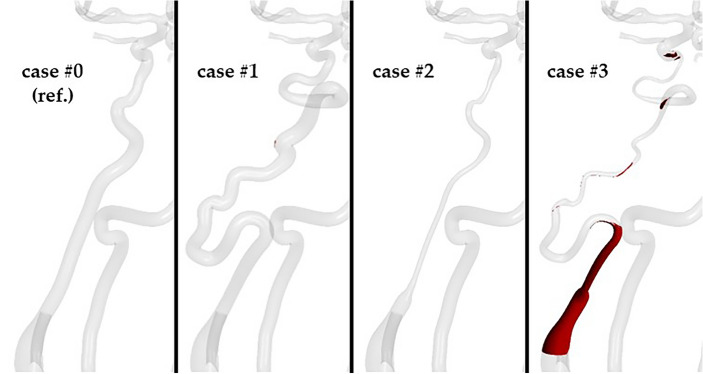
Regions of ‘old’ not-washed-out blood for each analyzed case studies at the final timestep of the simulation (*t* at 16.00 s).

In case #1, there are several regions of blood stagnation, particularly at bends in the vessel. In case #3, areas of low-velocity flow occur at the most proximal and distal portions of the ICA. Similar patterns are noted for regions of ‘old’ not-washed-out blood (Fig. 6, Supplemental Video S1). In proximity to these two regions are meaningful accumulations of ‘old blood’ that could start clotting.

**Figure 7:**
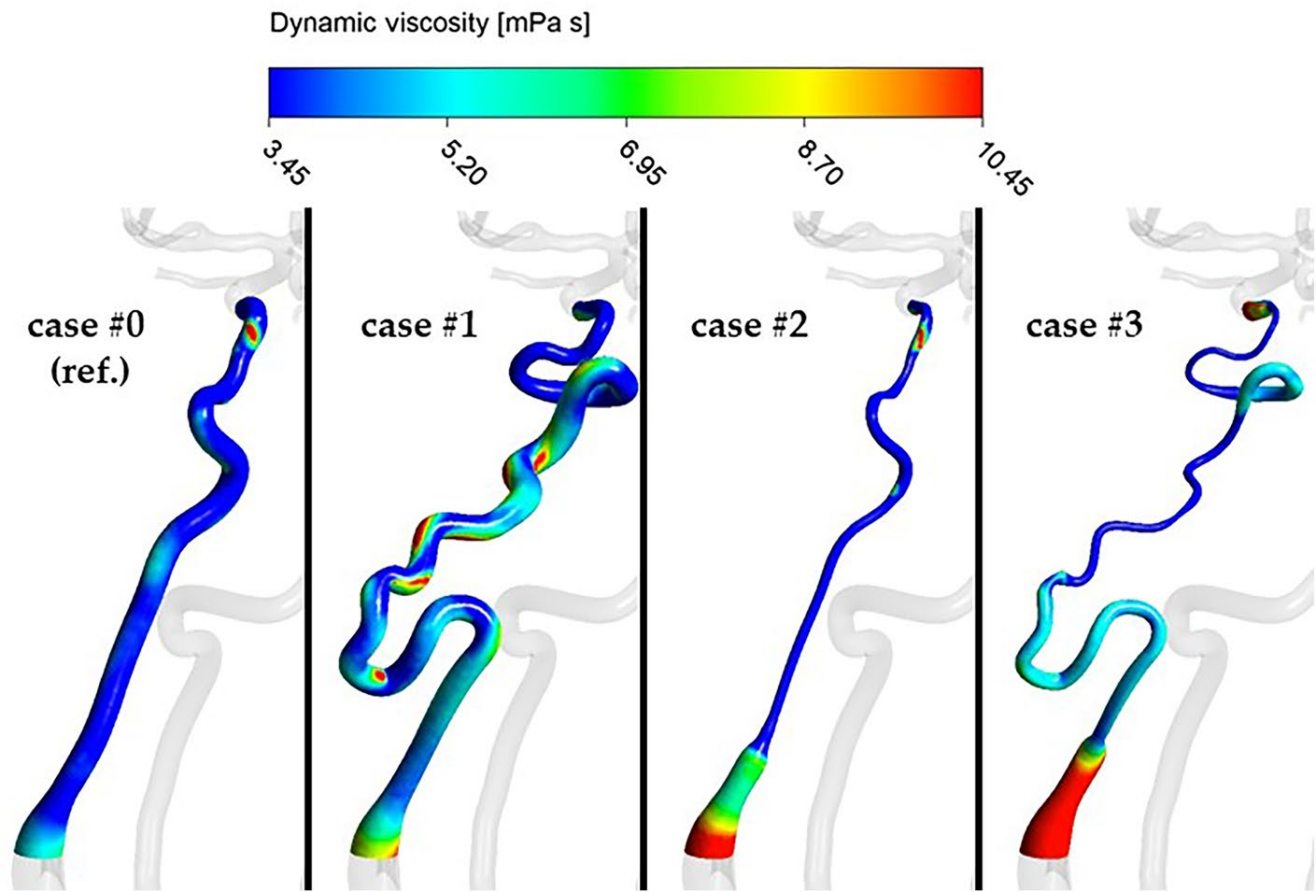
Distribution of dynamic viscosity in the region of left ICA at the final timestep of the simulation (*t* at 16.00 s).

Area-averaged blood viscosity for the last simulated timestep showed the same trend as the other parameters, with the highest increases noted for PHACES syndrome and the smallest increases for the vessel of normal diameter and high tortuosity (77.5% and 24.5%, respectively). Maximal dynamic viscosity demonstrated a similar pattern to area-averaged blood viscosity, with the exception of case #2 in which viscosity was reduced by 9.6%. Within the artery characterized by PHACES syndrome, viscosity reached 39.00 mPa s which is over 10 times higher than the standard value (3.45 mPa s). Figure 7 and Supplemental Video S2 depict a qualitative comparison of blood viscosity at the walls of the left ICA at the final timestep of the simulation (*t* at 16.00 s).

Varying topology of the blood vessel resulted in completely different distributions and local concentrations of high viscosity areas. In case #1 there were several regions of elevated viscosity along entire length of left ICA, generally in close proximity to the distal wall at arterial bendings. These regions corresponded to areas of low-velocity flow presented in Fig. 5. In the hypoplastic arteries (cases #2 and 3), just two regions of elevated viscosity were visible—at the distal ICA (just below ophthalmic artery) and the proximal ICA (just proximal to the hypoplastic segment). In case #3, blood viscosity increased substantially throughout a large segment of the proximal part of ICA, thereby conferring even greater thrombogenicity.

**Figure 8:**
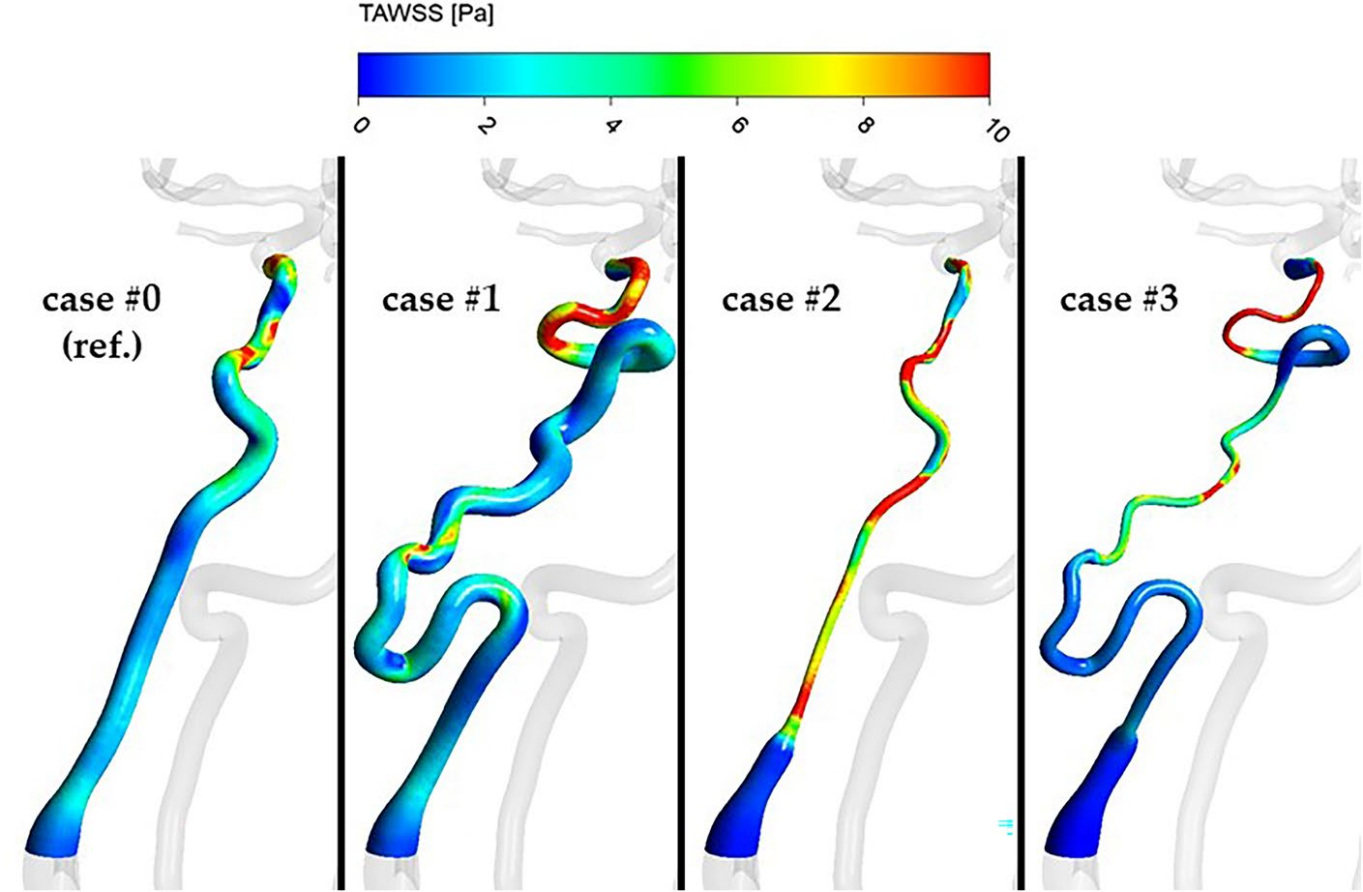
Distribution of TAWSS in the region of left ICA.

Quantitative and qualitative comparisons of stress-related parameters (pressure, WSS, TAWSS and OSI) are presented in Table 7 and Fig. 8, respectively.

**Table 7:** Values of the chosen hemodynamic parameters at the wall of left ICA (syndrome-affected).

Parameter		Case #0 (ref.)	Case #1	Case #2	Case #3
Systole peak (t at 15.52 s)	Area-averaged WSS [Pa]	3.02	3.41 (12.7%)	6.10 (101.9%)	2.49 (− 17.6%)
	Area-averaged pressure [Pa]	13,637	14,069 (3.2%)	12,085 (− 11.4%)	11,678 (− 14.4%)
Late diastole (t at 16.00 s)	Area-averaged WSS [Pa]	1.65	1.06 (− 35.8%)	2.97 (80.7%)	1.27 (− 23.0%)
	Area-averaged pressure [Pa]	9758	8483 (− 13.1%)	9261 (− 5.1%)	9176 (− 6.0%)
Cycle-averaged	Area-averaged TAWSS [Pa]	2.98	2.91 (− 2.3%)	5.46 (83.2%)	2.32 (− 22.1%)
	Area-averaged OSI [–]	0.156	0.176 (12.8%)	0.145 (− 7.1%)	0.145 (− 7.1%)

Changes in pressure/shear stress seem to vary for systole and diastole as well as between case studies, whereas for flow intensity, blood stagnation and relative ‘old blood’ area, PHACES syndrome had the greatest changes from the reference model, while solely high tortuosity had the least. We concluded that: in case #1, TAWSS remained nearly the same since shear rate, which can be associated with velocity gradient, was almost unaffected by change in artery topology. This was supported by the flow intensity analysis in which case #1 resulted in the smallest flow intensity reduction, a similar velocity gradient to the reference case, and almost no difference in TAWSS. Case #2 demonstrated a large increase in TAWSS due to high velocity gradients secondary to a very small cross section of the hypoplastic artery, despite significant reduction in flow intensity. According to the basics of fluid mechanics, the higher velocity gradient, the higher shear stresses. Case #3 had a reduction of TAWSS since the mutual influence of hypoplasticity and high tortuosity led to a drastic decrease of blood flow through the ICA resulting in a low velocity gradients despite the presence of a narrow arterial lumen.

## Discussion

In this retrospective study of children with PHACES syndrome utilizing a computational fluid dynamics model, we found that severe tortuosity and vessel hypoplasia were the most predictive factors for CVA.

PHACES syndrome is a rare condition observed in 2% to 3% of infantile hemangioma cases, with over 300 published cases. The precise incidence of CVA in PHACES is unknown and remains poorly understood^9^. The average age at the time of stroke is estimated to be 8.8 months^8^. However, reports vary; Siegel et al. reported on 22 patients with PHACES syndrome and arterial ischemic stroke and noted that 15 had an acute presentation at a mean age of 13.6 months. Burrows et al. reported progressive arteriopathy and cerebral infarction in four of seven children with acquired neurologic symptoms, all before the age of 18 months^11^. In our study, the median age for PHACES patients at the time of CVA was 13.5 months. Clinical symptoms were transient for all patients, without any persistent neurological deficits at 24-month follow-up. There were no instances of hemorrhagic stroke or death.

Several risk factors for CVA based on MRA findings have been previously reported despite a lack of data-driven evidence^11^. Severe (greater than 75%) stenosis or lack of visualization of a main cerebral artery with no evidence of collateral circulation were considered high-risk for CVA, whereas stenosis lower than 75% or lack of visualization of a main cerebral artery with collateral circulation, hypoplasia, dysplasia, aberrant origin and course of main vessels, aberrant subclavian artery and persistence of embryonic arteries were considered standard risk of CVA^22^.

Hess et al., showed that dysgenesis and abnormal vascular origin and course of the cervical and cerebral arteries, and a narrowing and lack of visualization of the affected vessel were associated with increased CVA risk^23^. Siegel et al., described a correlation between CVA and either a lack of visualization or narrowing of at least one main cerebral vessel in 19 of 22 cases. In 15 cases, more than 2 vessels were affected, and in 13 patients, there were associated aortic arch malformations^9^.

Garzon et al. differentiated between low, intermediate, and high risks groups^3^. The low-risk findings included common anatomic findings such as persistence of the embryonic artery, an abnormal arterial origin or course, and variations in the circle of Willis, which are hemodynamically insignificant. The intermediate risk group was comprised of patients with non-stenotic dysgenesis and narrowing or occlusion of the arteries of the Circle of Willis or located in its vicinity, with no effects on hemodynamics. The high-risk group showed significant narrowing or occlusion of cerebral vessels that are proximal to or part of the Circle of Willis, resulting in isolated circulation; multiple stenoses that are associated with complex blood flow, potentially interfering with brain perfusion; and findings in the brain parenchyma that suggest chronic and silent ischemia. Patients with cerebrovascular stenosis and coarctation of the aorta are also considered to be at high risk for neurological ischemic events. Altogether, stenosis or severe narrowing of primary cerebral blood vessels and the presence of coarctation of the aorta seem to be the most significant factors.

To date, there are no studies that demonstrated the combinatorial CVA risk of hypoplasia with severe tortuosity. Our retrospective analysis of institutional data showed that severe tortuosity was the strongest predictor of CVA, with smaller contributions by hypoplasia, absence of at least one main cerebral artery, and the presence of persistent arteries. Therefore, we performed CFD analysis to verify our results and build a computational model of CVA risk in PHACES syndrome.

In the current literature regarding blood flow, two methods of Computational Fluid Dynamics (CFD) are commonly used for fluid flow analysis: traditional CFD with rigid arterial walls and FSI (Fluid–Structure Interaction) where walls are elastic. Both these methods have their applications which depend on the research objectives^24,25^. In the current study, we concluded that FSI has an advantage over rigid-wall CFD because it takes into account the interactions between blood flow and the vessel wall (two-way coupling). Therefore, we could include physiological vasomotion of the arteries which allowed us to capture more realistic behaviour of blood flow hemodynamics. With the FSI approach, the walls of the arteries dilated during the systole peak and they returned accumulated energy to sustain the forward flow of blood during diastole. Moreover, if we assumed rigid walls of the arteries, we would obtain physiologically incorrect values—if the vessel cannot expand, there would be no pressure damping nor reduction of flow velocity. Moreover, individuals with PHACES syndrome exhibit high vessel tortuosity, which results in more significant interactions between blood flow and the vessel wall as compared to that found in healthy individuals. For this reason, we deemed that FSI is the only model whose application can approximate the natural conditions of blood flow in individuals with PHACES syndrome.

Using CFD we demonstrated that blood stagnation was minimal in a control model of the ICA but was substantially increased in the setting of severe tortuosity and hypoplasia. This was supported by the finding that ‘old’ not-washed-out blood was the highest in PHACES syndrome model, and the dynamic viscosity was 10 times higher in PHACES model compared to the control model.

Investigations of any maximal parameter estimated with CFD tools are highly related to the numerical mesh properties, e.g., it could be present in just a single node of the entire 3D mesh. Therefore, maximal value of any parameter might not be the most optimal indicator for a comparative analysis. Therefore, we based our analysis on area-averaged values.

Our results show that the combination of both hypoplasia and severe tortuosity is much more likely to result in blood stagnation and thrombus formation relative to either of these variables in isolation. Some studies have linked blood clotting with regions of low velocity, i.e., stagnation zones (velocity lower than 0.01 m/s)^18,26,27^. Since blood stagnation is heavily dependent on the timestep of the cardiac cycle, the authors do emphasize that this parameter cannot be solely used to define regions prone to thrombosis. That is why blood stagnation analysis must be accompanied by investigations of blood washout phenomenon (‘old blood’ area/volume that remains in the domain at the end of each cardiac cycle) and blood viscosity. Our findings suggest that small blood clots might form near those regions and become thromboemboli when they can detach from the wall, leading to stroke.

For PHACES syndrome arterial vessels, a nearly two-fold increase in blood viscosity was observed. When blood velocity is relatively high, and consequently shear rates are low, cells start to aggregate which increases viscosity. While prior studies have shown that CVA is multifactorial and the presence of certain anatomic variants such as aortic coarctation increase the risk of stroke, our study is the first to show that hypoplasia plus severe tortuosity are sufficient for thrombus formation. This explains the high risk of CVA in PHACES syndrome and corresponds to our institutional findings.

FSI simulations of blood flow take into account the deformability of vessels. We performed analyses in the whole arterial brain circulation in order to determine how blood supply decreases in each brain region in PHACES syndrome. Blood delivered throughout an entire cardiac cycle dropped from 3.82 to 0.44 cm^3^ for the ICA affected by PHACES syndrome. We noted also the highest differences in left and right anterior lobe blood supply were in cases with PHACES syndrome. We observed that blood supply reduction to the left ICA resulted in an increase of blood delivery to the right ICA and bilateral VAs in a stepwise fashion. This proves that absence or morphological change in a vessel on one side could amplify the flow on the opposite side. Such phenomenon could not be observed if we limited our study to just PHACES-affected artery. Therefore, it proves a hypothesis that any change of the model topology can change the flow resistance across the investigated model, and consequently, blood supply. Therefore, in-silico investigations should not be limited to just single arteries.

The reconstructed large flexible models are able to simulate real flow more closely, and therefore provide higher quality results compared to small models with rigid walls. They provide a better understanding of blood flow phenomena by taking a global view of how local flow dynamics affects whole cerebral circulation. We consider this a major strength of our study. The blood distribution observed in our FSI analysis is in concordance with MRI perfusion studies. Decreased CBF corresponded to a major arterial territory in arterial spin-labeling MRI perfusion^12^.

To date, the association between propranolol, which is the first-line pharmacologic treatment agent for complicated infantile hemangiomas, and serious adverse effects or stroke has not been proven^22^. In our opinion, a prospective study with CVA risk-stratified clinical subgroups should be performed. Propranolol decreases blood pressure and velocity, and may amplify the blood stagnation affected by hypoplasia and severe tortuosity. We believe that patients with PHACES syndrome who have high-risk CVA factors should be undergo more frequent monitoring and potentially early antiplatelet or anticoagulant prophylaxis, especially for patients on propranolol.

The limitations of our study include its retrospective nature. It is crucial to consider that PHACES syndrome is a rare clinical condition (less than 300 individuals were enrolled in the PHACES Syndrome International Clinical Registry and Genetic Repository), potentially resulting in diminished statistical power of the employed tests, particularly in scenarios where the discernible differences are of a minimal magnitude. Consequently, we advocate for the implementation of additional pooled analyses to facilitate the derivation of more reliable results. Another limitation is its single-center design, and in the case of retrospective analyses, challenges arise from gaps or errors in the database, especially concerning medication histories and imaging studies. The lack of propranolol data in our study is another limitation which requires further research.

In terms of the conducted FSI analyses, we can distinguish two major limitations. The first one is related to the modelling of the vessels walls. Due to lack of patient-specific data, we had to assume the thickness of each artery based on the available referentials and medical personnel expertise. Measurements of the arterial walls thickness could not be performed on the patient-specific CTA scans since thickness is characterized by significantly lower dimension when compared to the standard resolution of the CTA voxel (approximately 0.6 mm in each direction). Therefore, arterial walls could not be seen nor reconstructed from CTA or MRA scans. The second limitation might be related to the viscosity model of blood. Since we did not have access to the patient-specific viscosity measurements, we assumed a model that has been already utilized in numerous CFD researches of blood flows. However, it is worth noting that viscosity not only varies among each patient, but it usually changes for each patient during the day (since viscosity relies on a diet, hydration, temporary physical condition of the organism, etc.).

## Conclusion

The combination of severe tortuosity and hypoplasia presents a high risk for CVA in PHACES patients due to blood stagnation and thrombus formation. Therefore, PHACES patients with these risk factors may require more frequent follow-up visits and may be considered for prophylactic anticoagulation.

### Supplementary Information

Supplementary Information 1.

Supplementary Video 1.

Supplementary Video 2.

## Data Availability

Detailed data are presented in supplementary material.
