# Supplementary material for: Numerical flow experiment for assessing predictors for cerebrovascular accidents in patients with PHACES syndrome
Source: Sci Rep. 2024 Mar 2;14:5161. doi: 10.1038/s41598-024-55345-6 (PMC10908848; doi:10.1038/s41598-024-55345-6)
Supplement: Supplementary file 1 — Supplementary Information 1. [file 41598_2024_55345_MOESM1_ESM.docx]

**Details regarding imaging protocols and in-silico analyses**

*Magnetic resonance angiography protocol*

3D time-of-flight (TOF) magnetic resonance angiography (MRA) protocol, with a FISP (Fast Imaging Steady Precession) sequence and magnetization transfer was utilized. Imaging parameters were: TR 39 ms, TE 7 ms, MX 256-512, and FOV 210-210 mm. Time acquisition was 10 mm 30 s. The axially acquired angiographic slices were reformatted with a multiplanar reconstruction program (MPR). Then millimetric raw data images were postprocessed with a maximum intensity projection (MIP) technique. MRAs were performed on the General Electric Discovery MR450, featuring the CinemaVision system.

*Computed tomography angiography protocol*

All CTAs were performed on the General Electric 64-Slice LightSpeed CT Scanner with slice thickness of 0.625 mm and increment of 0.5 mm using 3D software (RadiAnt DICOM Viewer – Medixant, Poland). Head and neck CT angiography included the intracranial compartment and extended down to the aortic arch.

*Modelling of the arterial lumen*

By limiting our simulations of blood flow to just a small portion of the systemic circuit (a hypoplastic artery characterized by PHACES syndrome), we would not be able to capture the important phenomena that may occur in other parts of the arterial system. Thus, we decided to perform in-silico investigations in a systemic circuit including the aorta, visceral arteries, lower and upper limb arteries, as well as cerebral vasculature and all afferent blood vessels.

Unfortunately, it was not possible to gather biomedical imaging data of such a large region for a single patient because she/he would be overexposed to harmful radiation. Therefore, we decided to generate entire model by combining several different geometries of smaller regions:

1. Patient-specific aortic arch with all major branches;
2. Patient-specific descending aorta with visceral arteries up to iliac arteries;
3. Patient-specific cerebral vasculature together with distal segments of the ICAs and VAs;
4. Idealized arteries of lower/upper limbs.

A vast majority of the arteries was obtained by analyzing and processing biomedical imaging data, however, depending on the geometry type, varied techniques of model preparation were used. Each step of the reconstruction process is described below.

*Reconstruction of the 1^st^ and 2^nd^ parts: aorta with main branches*

After loading angio-CT DICOM dataset for a single patient into a custom-developed software (*Anatomical Model Reconstructor*, AMR), we manually selected points located at the vessel border which were added to a local list of contour vertices. This procedure was repeated several times to generate numerous cross sections of the same artery at different points. Afterwards, we prepared a centreline of the given artery following the same procedure. Then, this centreline and all aforementioned contours were exported as standard text files, compatible with computer-aided design (CAD) software. We conducted the same steps for each artery of interest.

For the remainder of the reconstruction process we used SolidWorks to recreate a volumetric spatial model of the arterial system, based on circumferential contours and vessel pathways. We utilized a contour-based extrusion between consecutive contours (profiles-lofting algorithm). Smooth transition between each segment was obtained due to tangent or normal-to-cross-section constrains. Having repeated this procedure for all the contours, each separate volumetric object represented an individual artery. All arteries were merged into a single model utilizing a simple Boolean operation. At bifurcation regions, we inserted artificial roundings to generate a smooth, anatomically correct transition. **Supplementary Figure 1** presents a workflow chart together with the reconstructed model.


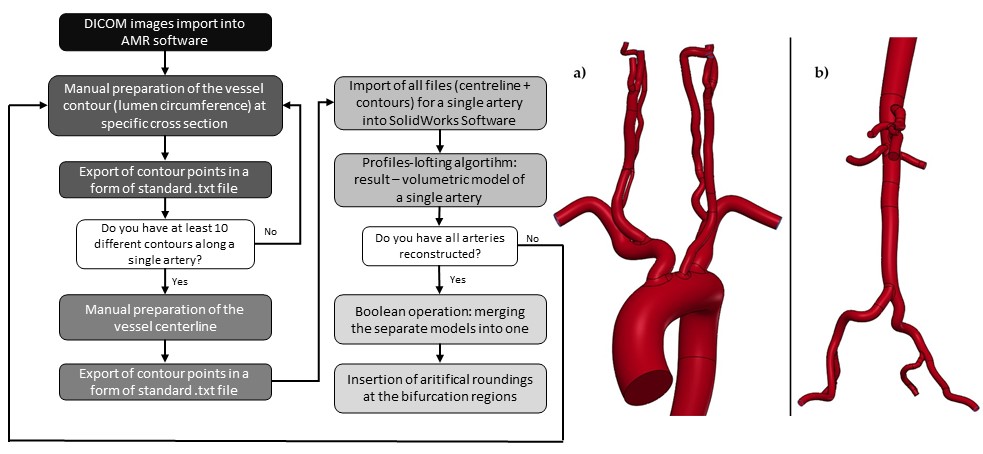


***Supplementary Figure 1.*** *Workflow chart together with final reconstructed model
of the patient-specific aorta with main branches*

*Reconstruction of the intracranial arteries plus distal ICAs and VAs*

We made a patient-specific model of the intracranial arteries and distal ICA/VA based on a patient with several intracranial aneurysms but otherwise normal cerebral vasculature. For reconstruction purposes, we utilized custom-developed AMR software for biomedical image segmentation to prepare a 3D binary mask representing the arterial lumen. A 3D model was then extracted from the binary mask with the use of image segmentation techniques (seed growing followed by manual corrections) and subjected to further processing. This included smoothing of the vessel walls while preserving the overall topology and clipping the distal-most parts of the arteries to create boundaries oriented normally to the flow channel. The prepared surface model was exported as a standard stereolithography format (STL) file. In order to combine this model with the model of the aortic arch using spatial alignment, we transformed it into an ANSYS SpaceClaim volumetric model.

First, the STL object was subjected to surface mesh repair (removal of self-intersecting facets, repair of non-manifold surfaces, etc.). Then, knowing that automatic Auto Skin method (which approximate small triangles forming the STL mesh with as few patches as possible) might produce several errors in the overall topology, we decided to split the model into several separate objects. Thus, for that purpose all bifurcations and all arteries were separated. Afterwards, Auto Skin method was applied to each segment separately. We automatically retrieved a majority of the intracranial model, but a few patches were missing which we generated using manual Skin methods. Each opening was capped with a next feature available in ANSYS SpaceClaim program and this model was exported in a format compatible with SolidWorks software. **Supplementary Figure 2** presents a workflow chart together with the reconstructed model.


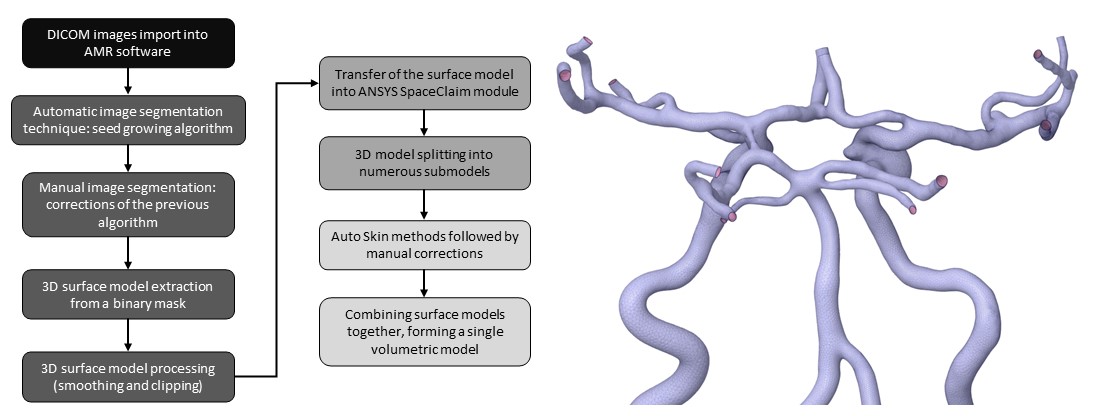


***Supplementary Figure 2.*** *Workflow chart together with final reconstructed model
of the patient-specific cerebral vasculature*

*Reconstruction of the 4^th^ part: arteries of the upper and lower limbs*

Due to lack of patient-specific data for the lower/upper limbs arteries, we created models based on previously published data using SolidWorks **[1]**. First, we imported anatomically correct images of upper/lower limb arteries pathways (in coronal and sagittal planes) and enlarged them to 1:1 scale of the human body. Subsequently, we manually generated a series of 3D points representing points along the given artery centreline. Then, a 3D spline passing through all points was created which represented the centreline of the vessel lumen. Using segment lengths and lumen diameters derived from the literature, we generated circular splines aligned normally to the centreline **[1]**. The next step was to utilize the profiles-lofting method with a constraint ‘along the centreline’ to generate a volumetric model of a single artery. All arteries were combined by a Boolean operation. Artificial roundings of either constant or varied radii were inserted at each bifurcation region. **Supplementary Figure 3** presents a workflow chart together with the reconstructed model.


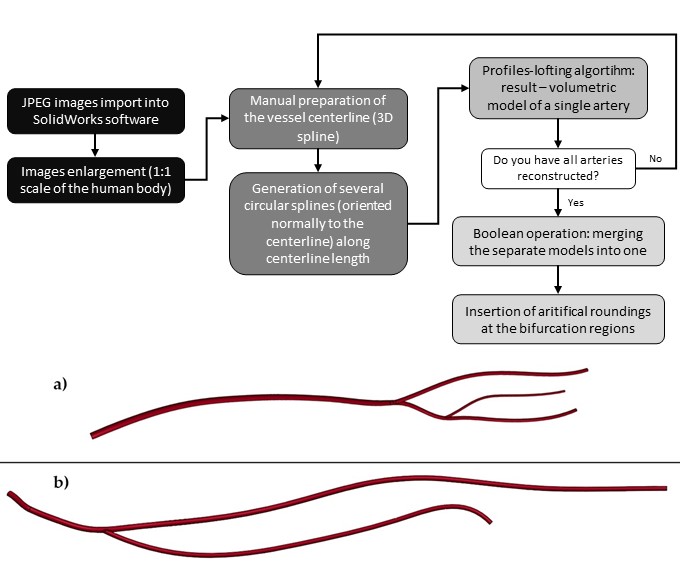


***Supplementary Figure 3.*** *Workflow chart together with final reconstructed model
of the upper/lower limb arteries*

*Modelling of porous bodies*

In CFD analyses of blood flow, flow directioning occurs due to pressure gradient between inlet and outlet cross sections. Pressure drops result from flow resistances. As pressure increases at an outlet cross section, less fluid will reach that region and more fluid will instead pass through the other channels with lower resistances. Therefore, we used porous bodies to provide artificial resistance behind the original outlet cross sections and control flow distribution across the entire systemic circuit. By modulating resistance in porous bodies we could obtain desired pressure drops or increases. The longer the porous body the higher the resistance, with the length equal to the circumference of the corresponding outlet cross section.

*Modelling of the vessel walls*

Fluid-structure interaction (FSI) simulations require having the structural (mechanical) model apart from traditional fluid domain. In our case, such a model should mimic the vessel walls that are characterized by vasomotion, expanding during systole (absorbing energy) and vasodilating during diastole (reverting to their initial size and returning part of the absorbed energy to the fluid to make it flow continually). We used SolidWorks to generate walls of anatomically correct thickness by offsetting surfaces of the volumetric object by a specific distance corresponding to the desired thickness. We encountered problems with offset surfaces overlapping or impossible-to-solve topology in areas where two arteries (or fragments of the same artery) were passing near each other in extremely close proximity. This was resolved by projecting 3D splines onto desired surfaces, remove overlapping regions and create a tangent connection between both structures. Moreover, traditional hole-filling with tangent constraints was usually insufficient, so one had to generate several guide curves and fill the interior with tangent patches.

*Modelling of the artery with PHACES syndrome*

Due to the absence of a patient-specific DICOM dataset of a patient with PHACES syndrome, we artificially generated the hypoplastic and tortuous ICA for this model. We loaded JPEG images of sagittal and coronal projections of the ICA changed with PHACES syndrome (images found in the online database: https://radiopaedia.org/cases/phace-syndrome-1) and used the same algorithm as previously described for the upper/lower limbs arteries. We next substituted the patient-specific ICA with this artificial one. Models in cases #2 and 3 were generated in the same manner (see **Supplementary Figure 4**). The entire model of the systemic circuit is presented in **Supplementary Figure 5**.


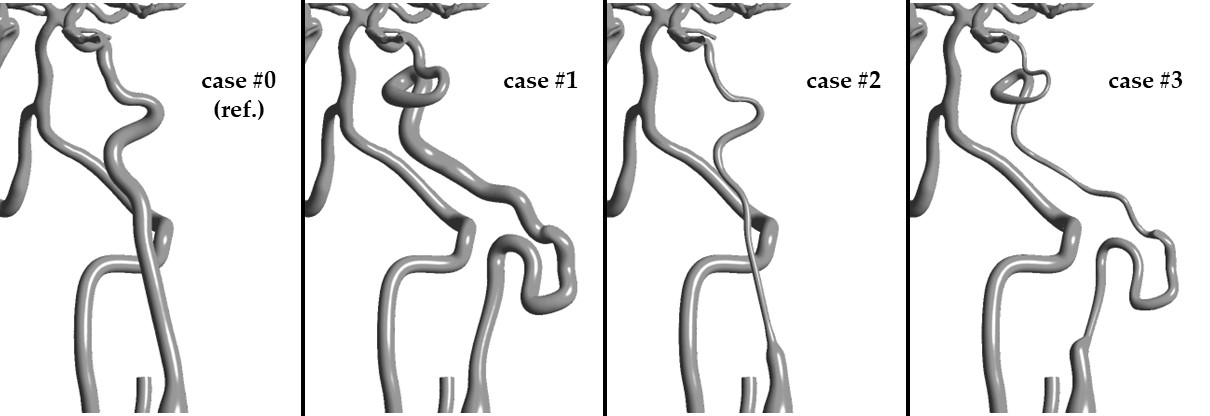


**Supplementary Figure 4.** Comparison of all geometries: case #0 – reference geometry (normal diameter, normal tortuosity); case #1 – left ICA with normal diameter and high tortuosity; case #2 – left ICA with hypoplasia, but normal tortuosity; case #3 – PHACES syndrome (hypoplasia + high tortuosity)


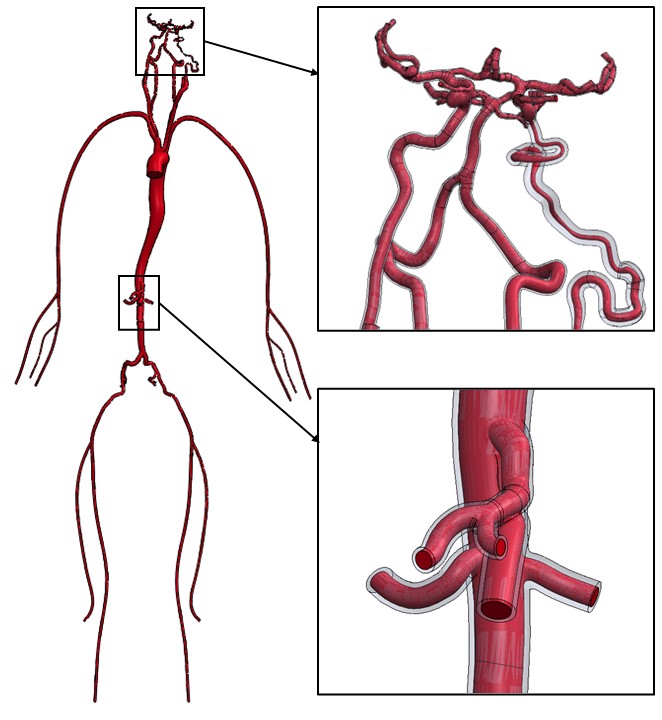


**Supplementary Figure 5***.* Geometry of the systemic circuit used during this study. PHACES syndrome is visible in one of the internal carotid arteries. Walls are made transparent, while vessel lumen is marked with a red color.

**Literature**

1. Reymond, P., Merenda, F., Perren, F., Rufenacht, D., & Stergiopulos, N. (2009). Validation of a one-dimensional model of the systemic arterial tree. American Journal of Physiology-Heart and Circulatory Physiology, 297(1), H208-H222.
